# First report of acanthocephalan parasite in wild-caught Asian vine snake (*Ahaetulla prasina*) in Indonesia

**DOI:** 10.14202/vetworld.2023.317-321

**Published:** 2023-02-17

**Authors:** Aditya Yudhana, Ratih Novita Praja, Ryanka Edila

**Affiliations:** 1 Veterinary Medicine Study Program, Department of Health and Life Sciences, School of Health and Life Sciences, Universitas Airlangga, Wijaya Kusuma Street 113, Banyuwangi, East Java, Indonesia; 2 Department of Veterinary Science, Division of Veterinary Parasitology, Faculty of Veterinary Medicine, Universitas Airlangga, Kampus C Mulyorejo Street, Surabaya, East Java, Indonesia; 3 Department of Veterinary Science, Division of Veterinary Microbiology, Faculty of Veterinary Medicine, Universitas Airlangga, Kampus C Mulyorejo Street, Surabaya, East Java, Indonesia; 4 Faculty of Veterinary Medicine, Universitas Airlangga, Kampus C Mulyorejo Street, Surabaya, East Java, Indonesia; 5 Royal Canin Veterinary Student Ambassador, Indonesia

**Keywords:** Acanthocephalan, *Ahaetulla prasina*, infectious disease, neglected disease

## Abstract

**Background and Aim:**

Exotic pet snakes are more susceptible to infection, especially parasitic helminths than wild-caught. There is no comprehensive report on the prevalence of acanthocephalan parasite infection in Indonesian snakes. Therefore, this study aimed to estimate the prevalence rate and to identify the acanthocephalan infection in wild-caught Asian vine snake (*Ahaetulla prasina*) from the Mojokerto District, East Java, Indonesia.

**Materials and Methods:**

A total of 60 snakes were collected from the local sellers in the Mojokerto District, East Java, Indonesia. Then, snakes were euthanized and necropsied to observe various predilections of acanthocephalan larval stage (cystacanth). Morphological identification of the cystacanth was conducted using the carmine staining method and microscopic examination.

**Results:**

Acanthocephalan infection was recorded with a high prevalence rate of 80.06%. A total of 696 cystacanths were examined from the muscle, subcutaneous tissues, and visceral with 32.90, 16.37, and 50.71% intensity rates, respectively.

**Conclusion:**

Acanthocephalan prevalence rate was recorded at 80.06% in this study. Constant disease monitoring is necessary, considering wild-caught Asian vine snakes were susceptible host and lack of data regarding parasitological surveys. Therefore, further studies are needed in new areas and various species of wild-caught snakes in Indonesia, because of the potential of parasitic helminth transmission between snake and other reptiles.

## Introduction

The number of wild-caught snakes kept as exotic pets have increased in many countries, including in Europe and Asia such as China, Thailand, and Indonesia. Wild-caught snakes are more susceptible to parasite infections than captive snakes. Venomous and non-venomous snakes have a similar risk of being infected by many parasites [1, 2]. Wild-caught reptiles are possibly infected by various parasites because of their natural prey and improper enclosure management, especially when kept in captivity. Moreover, snakes can suffer from prolonged dehydration when transported for long periods from the seller to breeders [3, 4]. High-stress conditions may lead to an increased number of parasitic infections and cause many pathologies in snakes. Moreover, parasitic diseases can kill snakes if the pathological condition is already in the chronic phase [5]. Most individuals who kept wild-caught snakes did not realize the risk of parasitic infection. Moreover, various parasitic helminths have a zoonotic potential and cause health problems in breeders. Thus, adequate examination by veterinarians also plays a key role in controlling parasitic helminth transmission from wild-caught snakes [6]. Parasitic helminths in snakes have been reported not only in the phylum Plathyhelminthes (class: Trematoda; Cestoda) and Nemathelminthes (class: Nematoda), and in the phylum *Acanthocephala* [7–11].

Acanthocephalans are known as thorny-headed worms or spiny-headed worms because of the unique characteristics of the hook-rows on the proboscis part, which is an important identification key for determining a genus or species [12]. Various insects and isopods usually play a role as intermediate hosts in their life cycle, whereas the definitive hosts vary according to the acanthocephalan species. In several species, the life cycle of acanthocephalans involves both invertebrates and vertebrates as intermediate hosts [13]. Reptiles and amphibians act as paratenic hosts for some acanthocephalan species possibly infected by ingesting various aquatic prey, such as fish [14–16]. Various species of predatory birds and mammals act as definitive hosts of acanthocephalans [17–19]. Acanthocephalans from the genera *Centrorhynchus* and *Sphaerechinorhynchus* infect and lead to pathological conditions in snakes [20, 21]. The larvae of *Centrorhynchus* spp. perform extraintestinal migration and infect tiger keelback snakes (*Rhabdophis tigrinus*) in Korea [20]. *Centrorhyn-chus sindhensis* infects Indian cobras (*Naja naja*), and the larval stage is known to have a predilection for the intestinal walls of snakes [22]. The cystacanth, as a fully developed larvae, infects painted green tree snakes (*Dendrelaphis punctulata*) in Australia [23]. *Sphaerechinorhynchus serpenticola* occurrence has been reported in wild-caught snakes, such as checkered keelback water snake (*Xenochropis piscator*) and painted bronzeback snake (*Dendrelaphis pictus*), in Indonesia [21, 24].

Although no reports of other species of snakes in Indonesia exist, accidental infection with acanthocephalans may occur, considering that some snakes eat the same prey in their natural habitat. Therefore, investigating the occurrence of acanthocephalan infections in other species of wild-caught snakes in Indonesia is necessary. To the best of our knowledge, no comprehensive data regarding acanthocephalans in Asian vine snake (*Ahaetulla prasina*) exist, even though these vine snakes are categorized as the favorite wild-caught snake, as vine snakes are kept as exotic pets by individuals or professional breeders.

This study aimed to estimate the prevalence of acanthocephalan infection in wild-caught *A. prasina* and to identify their morphological characteristics and predilections by parasitological examination.

## Materials and Methods

### Ethical approval

This study was conducted with prior permission from the local Wildlife Conservation Department in East Java Province. *Ahaetulla prasina* snakes are not categorized as endangered species, and their populations are widely distributed in Indonesia. This study was reviewed and approved by the Animal Care and Use Committee of the Faculty of Veterinary Medicine, Universitas Airlangga, Indonesia (vide No.1.KE.113.02.2021).

### Study period and location

Asian vine snakes were collected from local sellers from July to November 2021. Parasitological examinations were conducted at the Laboratory of Veterinary Parasitology, Division of Veterinary Parasitology, School of Health and Life Sciences, Universitas Airlangga, Banyuwangi, East Java Province, Indonesia.

### Snake samples identification

The Mojokerto District is a region of East Java Province, Indonesia (112.434084 longitudes and −7.472638 latitudes). A total of 60 snakes belonging to different age groups, such as hatchlings (0–70 cm), juveniles (71–95 cm), and adults (96–160 cm) were segregated by their length and investigated in this study. Asian vine snakes were categorized as wild-caught because no captive breeding farms for the snakes were available in the Mojokerto District.

### Parasitological examination

A total of 60 snakes were euthanized using ethyl-ether as inhalation anesthesia, skinned, and their muscular, visceral, and subcutaneous tissues were observed with the naked eye for cystacanth larvae. The number of parasites collected from each predilection was recorded to determine the rate of infection. Briefly, the helminth parasites were compressed between two slides and immersed in 70% ethanol for 24 h. The worm parasites were stained using a Semichon’s-acetocarmine solution for 30 min, washed, and decolored using 1% acid alcohol and 1% alkaline alcohol. Dehydration was then conducted using different concentrations of alcohol solution: 30% for 10 min, 50% for 15 min, 70% for 20 min, 90% for 30 min, and 95% for 30 min. Transparent parasite samples were immersed in xylol for 30 min and sealed with Entellan^®^ (Sigma-Aldrich, Singapore). The morphology of the parasite sample was examined using a binocular microscope (Olympus CX-23, Tokyo, Japan), and images were captured predominately in the anterior end part of the parasites for specific identification.

## Results

**Figure-1:**
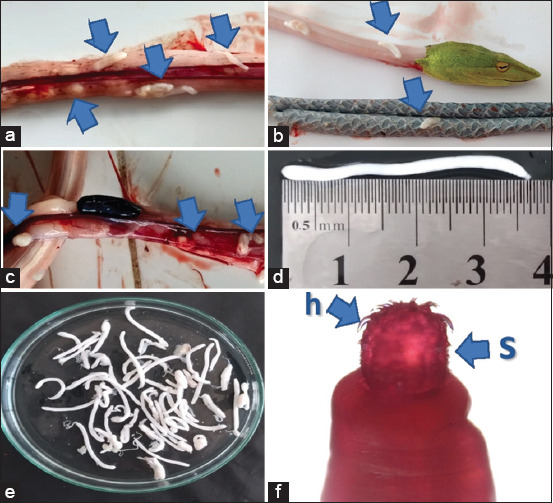
Acanthocephalan parasite in Asian vine snakes (*Ahaetulla prasina*). (a) Acanthocephalan in muscles (arrow), (b) subcutaneous tissue (arrows), (c) viscera (arrows). (d and e) Macroscopic appearance of acanthocephalan. (f) Photomicrographs of acanthocephalan anterior end illustrate proboscis with hooks (h arrow) and proboscis spine (s arrow) (Carmine staining, magnification, 100×) (arrow).

The prevalence rate of acanthocephalan parasites in Asian vine snakes from the Mojokerto District was 80.06%. All snake samples were wild-caught from several sub-districts and consisted of hatchlings, juveniles, and adults. In total, 696 acanthocephalan worms were successfully collected from 60 snakes. The number of acanthocephalans located in the muscle tissues (Figure-1a), subcutaneous tissues (Figure-1b), and visceral tissues (Figure-1c) was counted to investigate the distribution of acanthocephalans inside the snake body cavity. The intensity rates from each predilection were 32.90%, 16.37%, and 50.71%, respectively (Table-1). Macroscopically, the worms measured 2–4 cm in length and had a white cylindrical body shape (Figures-1d and e). Microscopic examination using Semichon’s acetocarmine staining method revealed that the parasites have an anterior morphology with several backwardly curving spines to attach themselves to the walls of their hosts (Figure-1f). The specimens were confirmed as acanthocephalan worms, based on specific spine and hook characteristics at the anterior end, in accordance with the previous findings by Audini *et al*. [21].

**Table-1:** Prevalence, intensity, and distribution of Acanthocephalan infection in Asian vine snakes (*Ahaetulla prasina*) from Mojokerto District, East Java Province, Indonesia.

Age of snakes	Number of samples (N)	Prevalence %	Intensity of *Acanthocephala*	No. of *Acanthocephala* in tissues		

				Muscles	Viscera	Subcutaneous tissues
Hatchling (0–70 cm)	6	83.3	34	15	9	10
Juvenile (71–95 cm)	39	76.9	530	176	78	276
Adult (96–160 cm)	15	80	132	38	27	67
Total	60	80.06	696	229	114	353

## Discussion

This study aimed to estimate the prevalence of acanthocephalan infection in wild-caught *A. prasina* and to identify their morphological characteristics and predilections based on parasitological examination methods. The life cycle and transmission pathways of parasitic helminths are fundamental aspects of veterinary medicine when focusing on wild animal hosts. Our findings represent the first report of an acanthocephalan infection in *A. prasina* which acts as a paratenic host for acanthocephalan parasites. The majority of definitive acanthocephalan hosts include carnivorous mammals and predatory birds [18, 19, 25]. Of all reptiles, snakes were categorized as the most frequent paratenic hosts, with varying rates of infection. However, further studies are needed to understand the complete life cycle, geographical distribution, and additional sampling efforts to comprehensively understand acanthocephalan transmission among snakes as their potential paratenic hosts [26]. Identification of acanthocephalan species requires examination of their larval (cystacanth) and adult stages using specific methods such as scanning electron microscopy or molecular identification, which was not conducted in this study. The general life cycle of acanthocephalans begins with egg ingestion by an arthropod as their intermediate host, in which development to cystacanth occurs. When the definitive host ingests the infected arthropod, the cystacanth undergoes the excystation process, which leads to maturation into the adult stage. In another scenario, when a paratenic host ingests the infected arthropod, the cystacanth migrates from the digestive tract into the body cavity and undergoes encystation [27]. The previous studies have reported similar findings of the cystacanth stage infecting various species of reptiles, such as rainbow lizards (*Mabuya quinquetaeniata*), ameiva lizards (*Ameiva ameiva*), and coastal clawed geckos (*Gonatodes antillensis*). Moreover, the predilection of cystacanth has also been reported in various body cavities, including subcutaneous tissues, causing nodules as a pathological sign [26, 28, 29].

A previous study in Indonesia revealed the presence of cystacanth stage in the body cavity of a painted bronzeback tree snake (*D. pictus*) with severe clinical signs [24]. Moreover, *D. pictus* and *A. prasina* have similar body size and feeding behaviors. Thus, acanthocephalan parasite transmission may occur in both snakes. Another study in Indonesia reported acanthocephalan occurrence in the *X. piscator* snake, which is frequently used in meat and skin products [21]. Furthermore, the total samples were similar to those used in this study, which used a total of 60 snakes. The prevalence rates of acanthocephalan infection were markedly different. An acanthocephalan infection in *X. piscator* snakes was recorded at 26.67%. In contrast, 80.06% prevalence rate was recorded in this study, implying that *A. prasina* is more susceptible as a paratenic host compared to *X. piscator*. The high prevalence rate identified in this study may be due to the natural behavior of Asian vine snakes, which are categorized as arboreal. Moreover, Asian vine snakes have a small body size and predominantly ingest various insects as prey, whereas insects are categorized as potential intermediate hosts for acanthocephalans.

Snakes suffering from acanthocephalan infection (acanthocephaliasis) show several clinical signs and pathological changes. Common clinical signs of acanthocephaliasis in snakes include lack of appetite, lack of activity, and dehydration. Subcutaneous nodules in several body parts also occur as a frequent pathological condition [23]. Significant damage to the internal tissues occurs in the intestine due to penetration and perforation of the acanthocephalan larvae. Severe intestinal damage is caused by s spiny proboscis deeply attached to the intestinal mucosa [1, 22]. Enteritis occurs due to a large number of inflammatory cells, accompanied by bleeding on the intestinal wall. As the intestinal villi are completely damaged, the surrounding tissues become fibrous and homogenous, indicating organ malfunction [2]. Consequently, snakes face significant nutritional problems due to painful sensations and a lack of nutrient absorption in the mucosal part of the intestine [6]. Understanding the pathogenicity of particular snake species, in addition to providing data on the parasitic helminth diversity of wild-caught snakes are crucial to establish prevention and therapeutic methods. Moreover, proper parasitic control measures could reduce the mortality of wild-caught snakes kept in captivity [11].

Snakes play an important role in maintaining ecosystem sustainability through wildlife interactions. *Ahaetulla prasina* is widely distributed and frequently found in the western to eastern regions of Java Island, Indonesia, where it originates [30]. Various threats, such as illegal hunting, deforestation, environmental pollution, and parasitic diseases, can influence snake health and significantly reduce the snake population in their natural habitat [31, 32]. At present, parasitological studies of wild-caught snakes in Indonesia remain challenging because several species are protected by the national conservation law and a lack of interest because the majority of people still consider snakes as dangerous creatures [33]. However, Indonesia has over a hundred snake species susceptible to various parasites infection [10]. Preliminary findings in this study highlight the role of wild-caught snakes as a source of parasitic diseases and provide additional potential routes of acanthocephalan transmission in Indonesian wildlife. Prevention measures should be implemented because of the high prevalence of wild-caught *A. prasina* kept as exotic pets. Therefore, further comprehensive surveys are needed to provide a deeper understanding of acanthocephalan occurrence in various species of snakes and other reptiles, as preliminary data have been reported in this study.

## Conclusion

This study is the first comprehensive report of acanthocephalan infection in *A. prasina* in Indonesia, with a high prevalence rate of 80.06%. Because wild-caught snakes are considered infrequent hosts and exclusive data on risk factors are lacking, constant monitoring, which includes parasitological surveys of native snakes, is important. Therefore, to determine the precise occurrence of parasitic helminth species, further studies should include new study areas in Indonesia, such as several districts located in Central and West Java Province, where the diversity of parasitic helminths in wild-caught snakes is less known. In addition, further studies on wild-caught snakes that are frequently kept as exotic pets in Indonesia (i.e., *D. pictus*, *Homalopsis buccata*, *Fowlea melanzostus*, *Cylindrophis ruffus*, and *Gonyosoma oxycephalum*) should be a priority, considering the potential for parasitic helminth transmission among them and other species of reptiles.

## Authors’ Contributions

AY: Designed the study, conducted the surveys, data analysis, and wrote the manuscript. RNP and RE: Designed the study, parasitological examinations, and collection and identification of snakes. All the authors have read, reviewed, and approved the final manuscript.

## References

[1] Rataj A.V, Lindtner-Knific R, Vlahović K, Mavri U, Dovč A (2011). Parasites in pet reptiles. Acta Vet. Scand.

[2] Divers S.J, Stahl J.S ((2019)). Mader's Reptile and Amphibian Medicine and Surgery.

[3] Wolf D, Vrhovec M.G, Failing K, Rossier C, Hermosilla C, Pantchev N (2014). Diagnosis of gastrointestinal parasites in reptiles:Comparison of two coprological methods. Acta Vet. Scand.

[4] Hallinger M.J, Taubert A, Hermosilla C (2020). Occurrence of kalicephalus, *Strongyloides*, and *Rhabdias* nematodes as most common gastrointestinal parasites in captive snakes of German households and zoological gardens. Parasitol. Res.

[5] Nasiri V, Mobedi I, Dalimi A, Mirakabadi A.Z, Ghaffarifar F, Teymurzadeh S, Karmi G, Abdoili A, Paykari H (2014). A description of parasites from Iranian snakes. Exp. Parasitol.

[6] Doneley B, Monks D, Johnson R, Carmel B (2018). Reptile Medicine and Surgery in Clinical Practice.

[7] Mihalca A.D, Miclŭş V, Lefkaditis M (2010). Pulmonary lesions caused by the nematode *Rhabdias fuscovenosa* in a grass snake, *Natrix natrix*. J. Wildl. Dis.

[8] Kavitha K.T, Latha B.R, Sundar S.T.B, Jayathangaraj M.G, Kumar K.S, Sridhar R, Basit S.A (2014). Kalicephalus spp in a captive Russell's viper:A case report. J. Parasit. Dis.

[9] Yudhana A, Praja R.N, Supriyatno A (2019). The medical relevance of Spirometra tapeworm infection in Indonesian bronzeback snakes (*Dendrelaphis pictus*):A neglected zoonotic disease. Vet. World.

[10] Yudhana A, Praja R.N, Pratiwi A, Kartikasari A.M (2021). Sparganosis in wild-caught Javanese keelback water snakes(*Fowlea melanzostus*)from Indonesia. Indian J.

[11] Halan M, Kottferova L (2021). Parasitic helminths in snakes from the global legal trade. Helminthologia.

[12] Van Ha N, Amin O.M, Ngo H.D, Heckmann R.A (2018). Descriptions of acanthocephalans, *Cathayacanthus spinitruncatus* (*Rhadinorhynchidae*) male and *Pararhadinorhynchus magnus* nspp. (*Diplosentidae*), from marine fish of Vietnam, with notes on *Heterosentis holospinus* (*Arhythmacanthidae*). Parasite.

[13] Halajian A, Smales L.R, Tavakol S, Smit N.J, Luus-Powell W.J (2018). Checklist of acanthocephalan parasites of South Africa. ZooKeys.

[14] Gupta N, Gupta D.K, Singhal P (2015). Description of *Pallisentis* (*Brevitritospinus*) *punctati* nspp. (*Acanthocephala*:*Quadrigyridae*) from channa punctatus in Bareilly, Uttar Pradesh, India. Iran. J. Parasitol.

[15] Amin O.M, Heckmann R.A, Bannai M.A (2018). *Cavisoma magnum* (*Cavisomidae*), a unique Pacific acanthocephalan redescribed from an unusual host, *Mugil cephalus* (*Mugilidae*), in the Arabian Gulf, with notes on histopathology and metal analysis. Parasite.

[16] Ngamniyom A, Wongroj W (2021). Ultrastructure and elemental depositions of hooks in *Centrorhynchus* cf *aluconi* (*Acanthocephala*:*Polymorphida*). Songklanakarin J. Sci. Technol.

[17] Lunaschi L, Drago F (2010). A new species of *Centrorhynchus* (*Acanthocephala*, *Centrorhynchidae*) endoparasite of *Guira guira* (*Aves*, *Cuculidae*) from Argentina. Helminthologia.

[18] Komorová P, Špakulová M, Hurníková Z, Uhrín M (2015). Acanthocephalans of the genus *Centrorhynchus* (*Palaeacanthocephala*:*Centrorhynchidae*) of birds of prey (*Falconiformes*) and owls (*Strigiformes*) in Slovakia. Parasitol. Res.

[19] Richardson D.J, Smales L.R, Ghorbani M.N, Halajian A (2017). *Centrorhynchus* spp (*Acanthocephala*:*Centrorhynchidae*) (from stray dogs *Canis familiaris*) in Qom, Iran. Comp. Parasitol.

[20] Choi C.J, Lee H.J, Go J.H, Park Y.K, Chai J.Y, Seo M (2010). Extraintestinal migration of *Centrorhynchus* spp(*Acanthocephala*:*Centrorhynchidae*) in experimentally infected rats. Korean J. Parasitol.

[21] Audini I.S, Suwanti L.T, Koesdarto S, Poetranto E.D (2017). Acanthocephalan in *Xenochrophis piscator* snake in Sidoarjo Indonesia. KnE Life Sci.

[22] Khan A, Khatoon N, Bilqees F.M (2002). *Centrorhynchus sindhensis*, new species (*Acanthocephala*:Centrorhynchinae) from the snake (*Naja naja*) intestine. Pak. J. Zool.

[23] Hill A.G, Ladds P.W, Spratt D.M (2014). Acanthocephalan infection and sparganosis in a green tree snake (*Dendrelaphis punctulata*). Aust. Vet. J.

[24] Yudhana A, Praja R.N, Supriyanto A, Oktaviana V (2018). First report of acanthocephalan infection in painted bronzeback tree snake (*Dendrelaphis*
*pictus*). J. Vet. Parasitol.

[25] Santos E.G.N, Chame M, Chagas-Moutinho V.A, Santos C.P (2017). Morphology and molecular analysis of *Oncicola venezuelensis* (*Acanthocephala*:*Oligacanthorhynchidae*) from the ocelot *Leopardus pardalis* in Brazil. J. Helminthol.

[26] Dornburg A, Lamb A.D, Warren D, Watkins-Colwell G.J, Lewbart G.A, Flowers J (2019). Are geckos paratenic hosts for Caribbean Island acanthocephalans?Evidence from *Gonatodes antillensis* and a global review of squamate reptiles acting as transport hosts. Bull. Peabody Mus. Nat. Hist.

[27] Norval G, Goldberg S.R, Bursey C.R, Dieckmann S, Mao J.J (2012). A description of parasites from mountain wolf snakes, *Lycodon ruhstrati ruhstrati* (*Serpentes*:*Colubridae*), from two localities in western Taiwan. Asian J. Conserv. Biol.

[28] Macedo L.C, Melo F.T.D, Ávila-Pires T.C.S, Giese E.G, Santos J.N.D (2016). *Acanthocephala* larvae parasitizing *Ameiva ameiva*
*ameiva* (Linnaeus, 1758) (*Squamata*:*Teiidae*). Rev. Bras. Parasitol. Vet.

[29] Rabie S.A.H, El-Din Z M, El-Latif A, Mohamed N.I, El-Hussin O.F.A (2015). Description of some acanthocephalan species from some reptiles in Qena governorate. J. Pharm. Biol. Sci.

[30] Kurniawan N, Fathoni M, Fatchiyah F, Aulani A, Septiadi L, Smith E.N (2021). Composition, distribution, and habitat type of snakes in Java, with discussion on the impact of human-snake interactions during 2013–2019. Herpetol. Notes.

[31] Zając M, Wasyl D, Różycki M, Bilska-Zając E, Fafiński Z, Iwaniak W, Krajewska M, Hoszowski A, Konieczna O, Fafińska P, Szulowski K (2016). Free-living snakes as a source and possible vector of *Salmonella* spp. and parasites. Eur. J. Wildl. Res.

[32] Demkowska-Kutrzepa M, Studzińska M, Roczeń-Karczmarz M, Tomczuk K, Abbas Z, Różański P (2018). A review of the helminths co-introduced with *Trachemys scripta elegans*-a threat to European native turtle health. Amphibia Reptilia.

[33] Yuniasih D, Tejosukmono A, Heriyanto J (2020). Snakebite as a neglected tropical disease in Indonesia:A review. Int. J. Sci. Technol. Res.

